# Complete chloroplast genome of the mixotrophic chrysophyte *Poterioochromonas malhamensis* (Ochromonadales, Synurophyceae) from Van Lake in Eastern Anatolia

**DOI:** 10.1080/23802359.2021.1923416

**Published:** 2021-08-27

**Authors:** Romain Gastineau, Elif Yilmaz, Cüneyt Nadir Solak, Claude Lemieux, Monique Turmel, Andrzej Witkowski

**Affiliations:** a Institute of Marine and Environmental Sciences, University of Szczecin, Szczecin, Poland; b Department of Biology, Arts and Science Faculty, Dumlupinar University, Kütahya, Turkey; c Département de biochimie, de microbiologie et de bio-informatique, Institut de Biologie Intégrative et des Systèmes, Université Laval, Québec, Canada

**Keywords:** *Poterioochromonas malhamensis*, Chrysophyceae, Van Lake, Synurophyceae

## Abstract

We sequenced the chloroplast genome of *Poterioochromonas malhamensis* (Pringsheim) R.A.Andersen strain SZCZR2049, which originates from Van Lake in Turkey. This genome is 133,923 bp long, and like those currently available for six phototrophic chrysophytes, it displays a long, gene-rich inverted repeat and a very short single-copy region. Compared to its chrysophyte counterparts, the *P*. *malhamensis* inverted repeat differs noticeably in gene content and the whole genome is missing 11 protein-coding genes. The maximum likelihood phylogeny inferred from concatenated protein-coding genes positioned *P. malhamensis* among the chrysophytes *sensu lato* as sister to the clade containing the Synurales (Synurophyceae) and Chromulinales (Chrysophyceae).

*Poterioochromonas malhamensis* (Pringsheim) R.A.Andersen 2017, formerly described as *Ochromonas malhamensis* (Pringsheim 1952), is a mixotrophic flagellated chrysophyte that belongs to the Ochromonadales (Synurophyceae). Photosynthesis was lost multiple times in chrysophytes, prompting studies of the molecular events underlying the transition from phototrophy to heterotrophy (Graupner et al. 2018; Dorrell et al. 2019). Known for grazing on bacteria (Holen 1999), *P*. *malhamensis* is also grazing on other microalgae (Zhang et al. 1996; Zhang and Watanabe 2001), an attribute representing a major threat to massive outdoor production of commercially important microalgae (Ma et al. 2017, 2018). *P. malhamensis* is a freshwater species but it can adapt to different salinities (Kauss 1974). We isolated this alga from a benthic sample of Van Lake, a saline lake from Eastern Anatolia. Here, we describe the chloroplast genome of this isolate and compare it to those previously reported for six phototrophic chrysophytes: five members of the Synurales (Synurophyceae, GenBank accessions MH795128-MH795132) (Kim et al. 2019) and *Ochromonas* sp. CCMP1393 (Chromulinales, Chrysophyceae, GenBank accession KJ877675) (Ševčíková et al. 2015).

A benthic sample was collected at a latitude of 39° 56′ 7.992″ N; 42° 16′ 52.993″ E during the month of February 2020 and was used as inoculum to initiate growth of *P*. *malhamensis* under illumination in F/2 medium based on natural freshwater (20 ‰ salinity). A clonal culture of this alga was established and is currently maintained in the Szczecin Culture Collection (http://geocentrum.usz.edu.pl/en/szczecin-diatom-culture-collection-szcz/, contact: Dr Przemysław Dąbek, pdabek@usz.edu.pl) under the accession number SZCZR2049. DNA was extracted following Doyle and Doyle (1990) and sequenced on the DNBSEQ platform by the Beijing Genomics Institute in Shenzhen. A sample of the DNA preparation is kept at −20 °C at the University of Szczecin. A total of 40-million paired-end reads were assembled using SPAdes 3.14.0 (Bankevich et al. 2012). Following identification of chloroplast contigs, the complete chloroplast genome sequence was assembled using Consed (Gordon and Green 2013). Genes were identified as previously described (Turmel et al. 2017).

The *P. malhamensis* chloroplast genome (GenBank accession MW175522) is 133,923 bp long and as previously documented for other photosynthetic chrysophytes (Ševčíková et al. 2015; Kim et al. 2019), it displays two identical copies of long, gene-rich inverted repeat (IR) sequences that are separated from one another by single-copy regions of vastly unequal lengths. At 1,619 bp, the small single copy region (SSC) contains only 5 conserved genes (*psaD, rpl21, rpl27, trnM, trnS*) in addition to *orf133* (divergent *ycf54* sequence). The long single-copy region (LSC) is 69,530 bp long and codes for 87 conserved proteins, 3 hypothetical proteins, and 15 tRNAs. The IR is 31,387 bp long and contains 15 conserved protein-coding genes, 5 ORFs, 3 rRNA genes and 7 tRNA genes. Although they were found in conserved gene contexts in the IRs of all previously examined autotrophic chrysophytes, the *psaM, petJ,* and *rpl34* genes appear to be entirely missing from the *P. malhamensis* genome. On the other hand, the *P. malhamensis* IR exhibits *dnaK* and a block of 4 contiguous genes (*ilvB*, *rbcS*, *rbcL* and *rps4*) that are located in the LSC in the 6 compared chrysophytes. Note here that blastn analysis of *P. malhamensis* SZCZR2049 *rbcL* revealed 100% identity with those previously reported for other strains of *P. malhamensis* (GenBank: EF165169, MH643685 to MH643690, MH643692). With respect to gene content, *P. malhamensis* also differs from phototrophic chrysophytes by the absence of a number of additional genes (*acpP*, *atpE*, *ftrB*, *psbW*, *rpl29*, *tsf*, *ycf12* and *ycf36*) and by the occurrence of *rpoC2* gene as two separate ORFs (the portion corresponding to the 3′ coding region is annotated as *orf648*). Breakup of the chloroplast *rpoC2* into two ORFs has also been reported in green algae belonging to the core Trebouxiophyceae and Chlorophyceae (Turmel and Lemieux 2018). The *dnaB*, *syfB* and *cemA* genes, which were found to be specific to the Synurophyceae (Kim et al. 2019), are missing from *P. malhamensis*.

A maximum likelihood phylogeny was inferred from 107 chloroplast protein-coding genes of 9 taxa, including the two representatives of the Eustigmatophyceae (*Nanochloropsis gaditana* and *Vischeria stellata*) that were used as outgroup (Figure 1). Sequence alignments were performed using MAFFT (Katoh and Standley 2013) and variable regions were removed with trimAl (Capella-Gutierrez et al. 2009). The phylogenetic analysis was carried out using RAxML 8.0 (Stamatakis 2014) under the GTR + I + G model, with the best tree out of 100 being computed for 1000 bootstrap replicates. In this tree, *P. malhamensis* represents the deepest branching lineage in the strongly supported clade (100% bootstrap support) containing the chrysophytes *sensu lato*. The relationships observed for the Synurales and Chromulinales are in agreement with the chloroplast phylogenomic analysis reported by Kim et al. (2019).

**Figure 1.**
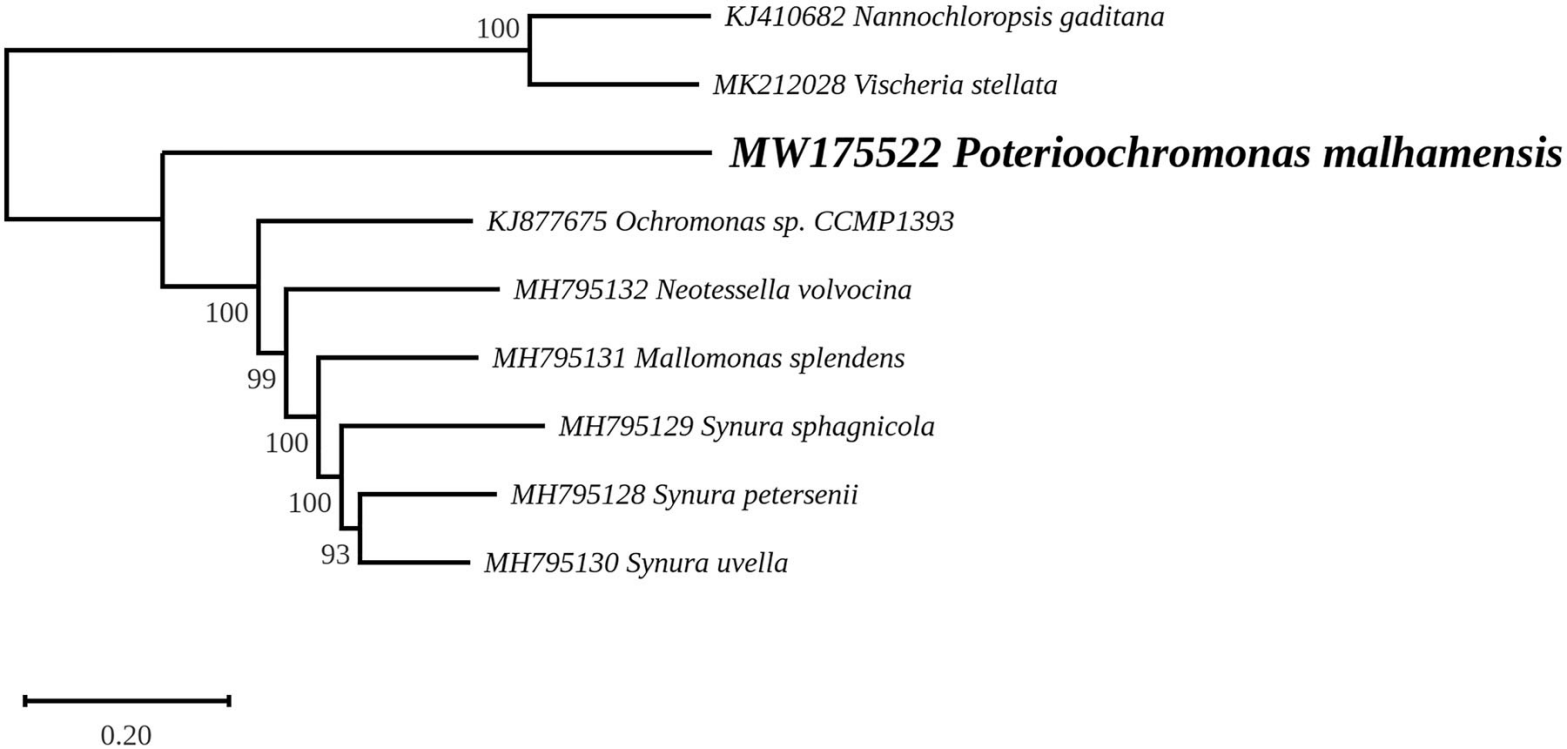
Maximum likelihood phylogeny obtained from 107 concatenated chloroplast protein-coding genes from *Poterioochromonas malhamensis* and 8 other taxa representing Chrysophyceae and Eustigmatophyceae. The best-scoring RAxML tree (log likelihood = −495241.595123) is presented.

## Data Availability

The authors confirm that the data supporting the findings of this study are openly available in GenBank of NCBI at (https://www.ncbi.nlm.nih.gov/) under the accession no. MW175522. The associated BioProject, SRA, and Bio-Sample numbers are PRJNA681133, SRR13155188, and SAMN16933430 respectively. The genome sequence is also available on Zenodo at: http://doi.org/10.5281/zenodo.4233311.
